# A 30-Year-Old Man With Rapid Floor-of-Mouth Swelling After Facial Trauma

**DOI:** 10.5811/cpcem.66219

**Published:** 2026-10-30

**Authors:** Mohammed Naveeth Imran, Surendar Ravipragasam, Shwetha Pujari Hemanthraj

**Affiliations:** Jawaharlal Institute of Postgraduate Medical Education & Research (JIPMER), Department of Emergency Medicine and Trauma, Puducherry, India

**Keywords:** traumatic pseudo-Ludwig angina, mandibular fracture, sublingual hematoma, Coleman sign, facial trauma

## Abstract

**Case Presentation:**

A 30-year-old man presented to the emergency department with severe blunt facial trauma following a fall from height. Examination revealed extensive floor-of-mouth ecchymosis and a large tense sublingual hematoma causing tongue elevation, dysarthria, and swelling mimicking Ludwig’s angina, producing a traumatic pseudo-Ludwig appearance with marked airway crowding. Three-dimensional computed tomography demonstrated severely displaced bilateral mandibular fractures. Owing to pronounced segmental instability with mobile dentoalveolar fragments, temporary interdental bridle wire stabilization was performed in the emergency department. Despite the dramatic anatomical findings, serial airway reassessments confirmed sustained airway stability, permitting delayed definitive open reduction and internal fixation.

**Discussion:**

Traumatic pseudo-Ludwig angina secondary to bilateral mandibular fractures is an uncommon presentation that may closely mimic life-threatening infectious floor-of-mouth swelling. Hemorrhage within the confined sublingual space can rapidly elevate and posteriorly displace the tongue, resulting in progressive airway compromise. This case highlights the importance of dynamic airway assessment, serial airway reassessment, and early multidisciplinary management, including temporary interdental bridle wire stabilization, in carefully selected patients with traumatic hemorrhagic floor-of-mouth emergencies.

## CASE PRESENTATION

A 30-year-old man presented to the emergency department with severe blunt facial trauma following a fall from height. On arrival, he was hemodynamically stable with vital signs as follows: blood pressure, 110/80 mm Hg; heart rate, 92 beats per minute; respiratory rate, 16 breaths per minute; and SpO_2_, 97% on room air. The primary trauma survey identified no other immediately life-threatening injuries requiring urgent intervention. Examination revealed lower facial swelling, facial abrasions, ecchymosis, and marked mandibular instability. Intraoral examination demonstrated extensive floor-of-mouth ecchymosis and a large tense sublingual hematoma causing tongue elevation, dysarthria, and marked airway crowding, closely mimicking Ludwig’s angina and producing a traumatic pseudo-Ludwig appearance (Image 1). Despite the dramatic anatomical findings, the patient maintained a patent airway, handled oral secretions without difficulty, and exhibited no stridor or respiratory distress.

Three-dimensional computed tomography demonstrated severely displaced bilateral mandibular fractures involving the parasymphysis and body-angle regions (Image 2). Owing to pronounced segmental instability with mobile dentoalveolar fragments, temporary interdental bridle wire stabilization was performed in the emergency department following consultation with the oral and maxillofacial surgery team (Image 3). The patient remained under close observation in the emergency department with serial airway reassessments, which confirmed sustained airway stability and partial resolution of the sublingual hematoma over the subsequent 24 hours. Following reassessment by the oral and maxillofacial surgery team, he was discharged from the emergency department with planned delayed definitive fixation.

## DISCUSSION

Traumatic pseudo-Ludwig angina secondary to mandibular fractures is an uncommon but potentially life-threatening presentation that closely mimics infectious Ludwig’s angina. Most reported cases of pseudo-Ludwig angina are associated with anticoagulation, coagulopathy, or spontaneous sublingual hematoma, whereas trauma-related cases remain exceedingly rare.^1^–^3^ Distinguishing traumatic pseudo-Ludwig angina from deep neck space infection is crucial because the underlying pathology is hemorrhagic rather than infectious; therefore, management prioritizes dynamic airway assessment, identification of the bleeding source, and timely hemorrhage control.

Hemorrhage within the confined sublingual space can rapidly elevate and posteriorly displace the tongue, resulting in dysarthria, dysphagia, drooling, and progressive airway compromise. Coleman sign, a bluish, tense swelling of the floor of the mouth caused by sublingual hemorrhage, is an important clinical clue to mandibular fracture and should alert emergency physicians to the possibility of an evolving airway emergency. Computed tomography is the imaging modality of choice for confirming mandibular fractures and delineating associated injuries.^3^,^5^

*CPC-EM Capsule*

What do we already know about this clinical entity?

*Infectious Ludwig’s angina is well known, but traumatic pseudo-Ludwig angina is an exceedingly rare mimic of deep neck infection.*

What is the major impact of the image(s)?

*Bilateral mandibular fractures produced a dramatic pseudo-Ludwig appearance from sublingual hemorrhage despite preserved airway physiology.*

How might this improve emergency medicine practice?

*Recognizing this traumatic mimic and using physiology-guided airway assessment may help avoid unnecessary airway escalation in stable patients.*

From an emergency medicine perspective, management should prioritize early recognition of dynamic airway compromise following the primary trauma assessment. Although marked tongue elevation and floor-of-mouth swelling may appear alarming, the decision to secure the airway should be guided by physiologic deterioration rather than anatomical appearance alone. Progressive stridor, inability to handle oral secretions, hypoxemia, worsening dysphonia, or rapidly expanding swelling warrant early definitive airway management with preparation for a surgical airway when indicated.^4^ In the absence of these features, carefully selected patients may be managed with close observation, serial airway reassessment, CT evaluation, and early oral and maxillofacial surgery consultation while definitive fracture management is coordinated.^5^

Despite marked tongue elevation and significant airway crowding, our patient remained clinically stable throughout repeated bedside airway assessments, allowing definitive airway intervention to be safely deferred. Temporary interdental bridle wire stabilization reduced movement of the displaced fracture segments, potentially limiting ongoing soft tissue injury while facilitating continued observation before definitive fixation. Although temporary stabilization is not a substitute for definitive airway protection or fracture fixation when clinically indicated, this case highlights the importance of repeated bedside airway evaluation, multidisciplinary collaboration, and timely temporary fracture stabilization in facilitating safe delayed definitive management in carefully selected patients with traumatic pseudo-Ludwig angina.^3^–^5^

## Figures and Tables

**Image 1:**
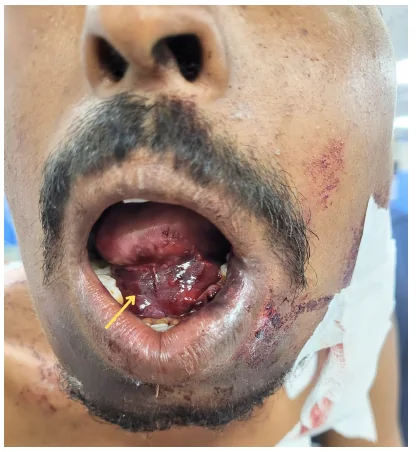
Extensive floor-of-mouth ecchymosis and massive sublingual hematoma causing tongue elevation and traumatic pseudo-Ludwig appearance (arrow).

**Image 2:**
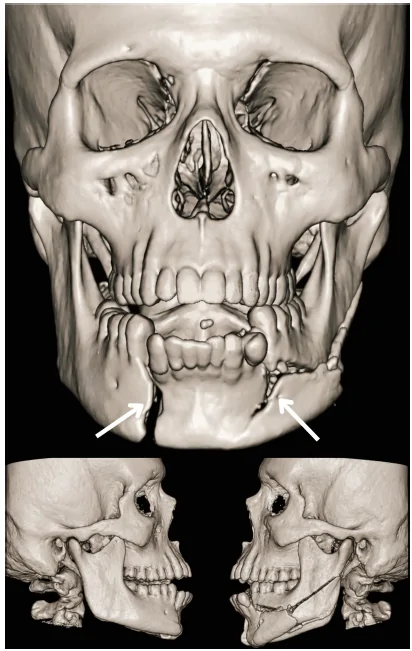
Three-dimensional computed tomography demonstrating severely displaced bilateral mandibular fractures involving the parasymphysis and body-angle regions (arrows).

**Image 3:**
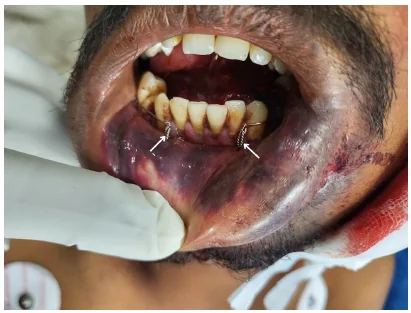
Temporary interdental bridle wire stabilization performed in the emergency department for mandibular segmental instability (arrows).

## References

[1] Ekin U, Hazari A, Alyassin N (2025). Successful management of pseudo-Ludwig angina from supratherapeutic warfarin use: a case report. Clin Pract Cases Emerg Med.

[2] Kellman RM, Losquadro WD (2008). Comprehensive airway management of patients with maxillofacial trauma. Craniomaxillofac Trauma Reconstr.

[3] Marin E, Fauconnier S, Bauters W (2020). Idiopathic spontaneous sublingual hematoma: a case report and literature review. Oral Maxillofac Surg.

[4] Piccini GB, Sfondrini D, Tomulescu SA (2026). Modern management of maxillofacial trauma in the emergency department. World J Emerg Med.

[5] Shukri FAA, Mohammad A, Johari AH (2026). Sublingual hematoma secondary to mandibular trauma mimicking Ludwig’s angina: a clinical conundrum. Univ Tor Med J.

